# Supplementary material for: Impact of the collaboration mechanism of PPP projects based on consumer participation: A system dynamics model of tripartite evolutionary game
Source: PLoS One. 2021 Sep 8;16(9):e0256304. doi: 10.1371/journal.pone.0256304 (PMC8425542; doi:10.1371/journal.pone.0256304)
Supplement: Attachment — Submitted filename: Review report (PONE-D-21-07314).docx [file pone.0256304.s001.docx]

**SUMMARY OF THE REVIEWER’S REPORT**

**The Evaluation of the paper titled:** “*Impact of the collaboration mechanism of PPP projects based on consumer participation: A system dynamics model of tripartite evolutionary game.”*

| Category | Remarks |
| --- | --- |
| ***Abstract*** | - The abstract is loosely written. For example, in the fourth line of the abstract you spoke only about the government perspective but did not mention any thing about the private sector or consumer’s perspective, although you are speaking about a tripartite game (Why?). - Moreover, it is not as informative as expected. A standard abstract must present, without leaving any doubt, the objective of the paper precisely; source of data (which is not present in your abstract) and analytical approach used; key findings and any policy implication and recommendations. |
| ***Introduction*** | - The arguments are fairly presented but the statement that justifies the study does not come clearly (i.e. Why did you started this research?). - The introduction does not precisely craft the research problem tackled and does not show how the problem is taken care. - The terms used are not clearly identified even in the lit. Review. For instance, what do you mean by social capital and public. No definitions are provided for them. If social capital refers to private capital, you have to reconsider the usage of this term because it will be confusing. - Moreover, you used the term private capital latter in the literature review instead of social capital. - What do you mean by giving the government the role of the regulator? What does a regulator do? - Sometimes you write PPP model and in other parts PPP mode - There is no consistency in the term usage. - In Fig. (1) You used private capital inside the figure and social capital in the title of the figure (i.e. no consistency). - The sentence of the results in the last paragraph of the introduction is incomplete and not clear - The research hypotheses’ are not mentioned in the introduction or clear in the literature review. Their first mention is in the third section of the research. |
| ***Literature review and critical***  ***analysis of theories, practices or***  ***commentary focusing on existing***  ***documents*** | - The study lacks clear description of the literature review: - What I am missing is a description of the review. Did you conduct a systematic literature review? Which years? Key words? What was the literature you found? - What is the relation between evolutionary theory and tripartite interests? This is not clear - Can you better describe how you came to your major variables? You have them from the literature review, but how was literature screened to derive these factors. |
| ***Methodology and scope of work*** | The analytical design is O.K. |
| ***Results*** | The author has poorly discussed the results of the paper. One would expect to find the previous empirical work enriching the discussions of the results, but unfortunately, that has not been done. |
| ***Conclusion and Recommendation*** | - The part of the recommendations is rather short, maybe you can strengthen that part in a way which really show the implication of the findings more clearly |
| ***Referencing*** | Referencing is O.K |
| Overall, the paper is readable, but there are still many typing errors. Sometimes words are missing or the word order is confusing. Therefore, I would recommend language editing and proof reading. | |
