# Supplementary material for: Impact of the collaboration mechanism of PPP projects based on consumer participation: A system dynamics model of tripartite evolutionary game
Source: PLoS One. 2021 Sep 8;16(9):e0256304. doi: 10.1371/journal.pone.0256304 (PMC8425542; doi:10.1371/journal.pone.0256304)
Supplement: Attachment — Submitted filename: Response to Reviewer #2.docx [file pone.0256304.s002.docx]

**Cover letter**

Dear reviewer:

We are very sorry that we did not pay attention to your suggestion for revision before.

Thank you very much for your evaluation and comments on our paper “*Impact of the collaboration mechanism of PPP projects based on consumer participation: A system dynamics model of tripartite evolutionary game*”. We have revised the manuscript according to your kind advices. We sincerely hope this manuscript will be finally acceptable to be published on PLOS ONE.

Best regards

Sincerely yours

Dr. WEILIU

Response to the reviewer’s comments

Comment 1: Need to better highlight the novelty of the study in the introduction.

Response: Thank for your suggestion. We have added the description of the novelty the last paragraph in page 3 and the second paragraph in page 4.

Comment 2: Cleary define motivations for your research.

Response: Thank for your suggestion. We have added the description why and how to research by this paper in the last two paragraphs introduction.

Comment 3: Literature review – A more comprehensive literature review is highly recommended, particularly published papers in recent years. You should introduce more recent papers. Moreover, at the end of the literature review based on current gaps provided in the literature, the contributions of this manuscript must be clarified better.

Response: Thank for your suggestion. We have modified the content from the third paragraph in page 3 to the third paragraph in page 4. We search more papers about PPP mode and evolutionary game. And it expresses the gaps in recent years.

Comment 4: Validation should be better organized.

Response: Thank for your suggestion. The purpose of the validation analysis in this paper is that verify the behavioral validity. Validation analysis is based on the subjective setting of parameters in accordance with the realistic system. The main purpose is to verify whether the change of different parameters has influence on the target variable.

Comment 5: Conclusion should be extended by highlighting the study novelty. More future directions should be presented.

Response: Thank for your suggestion. We have added the conditions and role of consumer participation, which is the novelty of this paper in content 5.1 conclusion. Future directions have been expressed in 5.3 Limitations and future work.
