# Supplementary material for: Impact of the collaboration mechanism of PPP projects based on consumer participation: A system dynamics model of tripartite evolutionary game
Source: PLoS One. 2021 Sep 8;16(9):e0256304. doi: 10.1371/journal.pone.0256304 (PMC8425542; doi:10.1371/journal.pone.0256304)
Supplement: Attachment — Submitted filename: Response to Reviewer #1.docx [file pone.0256304.s003.docx]

**Cover letter**

Dear reviewer:

Thank you very much for your evaluation and comments on our paper “*Impact of the collaboration mechanism of PPP projects based on consumer participation: A system dynamics model of tripartite evolutionary game*”. We have revised the manuscript according to your kind advices. We sincerely hope this manuscript will be finally acceptable to be published on PLOS ONE.

Best regards

Sincerely yours

Dr. WEILIU

Response to the reviewer’s comments

Comment 1: The definition of PPP in the first paragraph of the introduction (P.34), look as if it it is inserted by mistake, there is no fluency of ideas in the paragraph. Moreover, no reference is mentioned for this definition.

Response: Thank for your suggestion. We have optimized the fluency of the first paragraph in Introduction. We marked a reference to the PPP definition.

Comment 2: There are some grammatical mistakes in the text (e.g. 'consumers is' in the 2nd line in the 1st paragraph of the conclusion p.54) and sometimes sentence are written in a way that is not clear and ambiguous (especially the new parts in the discussion and conclusion). So, I would recommend revision and proofreading for the article.

Response: Thank for your suggestion. We have revised and proofread for the whole article.
